# Revisiting traditional practices in the management of acute uncomplicated diverticulitis: a call for change

**DOI:** 10.1007/s00384-024-04648-1

**Published:** 2024-05-21

**Authors:** Syed Mohammad Nabeel Aamir, Safa Nasir, Syeda Aisha Waqar

**Affiliations:** 1 PGY2 Surgical unit-1, Department of Surgery, Abbasi Shaheed Hospital, Karachi, Pakistan; 2 Department of Internal Medicine, Aga Khan University Hospital, Karachi, Pakistan; 3 Department of Plastic Surgery, Abbasi Shaheed Hospital, Karachi, Pakistan

We read with great interest the article ‘Systematic review and meta-analysis of the management of acute uncomplicated diverticulitis: time to change traditional practice’, written by Ali Yasen Mohamedahmed et al. and published in the ‘*International Journal of Colorectal Disease*’ which showed that observation-only treatment is feasible and safe in selected clinically stable patients with uncomplicated acute diverticulitis (Hinchey 1a classification), providing better outcomes including decreased length of hospital stay. Moreover, the outpatient (OP) approach in treating patients with Hinchey 1a acute diverticulitis is comparable to inpatient (IP) management. Future high-quality randomised controlled studies are needed to understand the outcomes of the non-antibiotic (NABX) approach used in an OP setting in managing patients with uncomplicated acute diverticulitis [1]. 

Regardless of these conclusions holding true, there exist various other confounding factors that may potentially influence the study results and must be considered in order to eliminate any possible skew. Diverticulitis is a relatively common disease of the elderly population with a multitude of causes including, but not limited to, genetics. The specific cause leading to diverticular disease gives clues regarding disease progression, remission, recurrence, rate of complication etc. Diverticulitis with a genetic proponent behind it may recur throughout the patient’s life; however, those having a dietary or sedentary lifestyle-related cause may experience complete remission and a relatively benign disease course. Since specific risk factors and causes leading to diverticular disease ultimately determine disease progression and outcome, patients should be risk stratified accordingly in order to eliminate any confounding factors obscured from the research findings [2, 3].

Furthermore, inflammatory bowel disease (IBD), especially Crohn’s disease, acts as a major contributor to the development of colonic diverticula, with the latter being a complication of the former. Patients afflicted with the said disease experience recurrent diverticula and increased rate of complication development including perforation, abscesses and fistulae. The study article fails to describe whether the patient population being studied were inquired regarding an existing history of IBD or if they are concurrently on chronic immunosuppression as the presence of any of these could potentially influence complication development and disease recurrence, hence obscuring the true results [4, 5].

In addition to the points described above, acute diverticulitis is divided on the basis of severity into mild, moderate and severe types. Such categorisation aids in determining the choice of effective antibiotics to be administered to the patient. For mild (Hinchey 1a classification) diverticulitis, guidelines suggest the treatment to be on outpatient basis. A good first-choice drug is trimethoprim/sulfamethoxazole, double-strength, given twice a day for 7 to 10 days. Another good first-choice drug combination is oral ciprofloxacin, 500 mg twice a day. Oral metronidazole, 500 mg every 6 h, is added to either regimen for anaerobic gram-negative bacilli with each regimen continued for 7 to 10 days. An alternate drug combination is amoxicillin/potassium clavulanate 500 mg/125 mg three times a day for 7 to 10 days. The oral antibiotics should be started as soon as possible after confirmation of the diagnosis. The primary article fails to describe if these guidelines were uniformly and meticulously followed, whether there was any deviation from the guidelines and which specific antibiotic therapies were administered. Furthermore, for a more pristine approach, patient stratification into separate treatment arms is required and individual response to antibiotics studied separately [6].

When it comes to the observation and conservative approach, there also exist various modalities to choose from with dietary and lifestyle changes, laxatives, fluid therapy, probiotics being a few. The conservative management targets diverticula developing via specific risk factors and eliminating the cause at root level. Thus, every patient will require individually tailored therapy based on their specific risk factors. The NABX arm should further be stratified according to these modalities to reveal any findings obscured by confounding [6].

When it comes to diverticulitis, differentiating between complicated and uncomplicated cases plays a key role in tailoring the management towards the patient population. Since uncomplicated diverticulitis is the primary focus of the study being conducted, it is imperative that a sharp demarcation line be set to separate the complicated cases from the uncomplicated ones. A novel approach is by using biomarkers to aid in the differentiation. CA-125, in particular, has shown great promise in shrewdly distinguishing between the two. Hence, a more conservative approach would adopt the usage of this biomarker to further strengthen the inclusion and exclusion criteria and prevent any selection bias [7].

In summary, regardless of the factors described by the authors holding true, there exist potential confounders within those parameters that may, effortlessly, be eliminated by simple stratification of the patient population. These factors are readily available and can be an inexpensive addition to the risk stratification model outlined the by the authors.

## Data Availability

No datasets were generated or analysed during the current study.
